# Utilization of cervical cancer screening and its associated factors among women of child-bearing age in Mangochi district, Malawi: a facility-based cross-sectional study

**DOI:** 10.1186/s12905-023-02472-3

**Published:** 2023-06-24

**Authors:** Felistas Mpachika-Mfipa, Lucy Ida Kululanga, Dumisani Mfipa, Abigail Kazembe

**Affiliations:** 1 Department of Nursing, Phalombe District Health Office, P. O. Box 79, Phalombe, Malawi; 2 School of Nursing, Department of Community Health Nursing, Kamuzu University of Health Sciences, Private Bag 360, Blantyre, Malawi; 3 C/o Phalombe District Hospital, P.O. Box 79, Phalombe, Malawi; 4 School of Maternal, Neonatal and Reproductive Health, Department of Midwifery, Kamuzu University of Health Sciences, Private Bag 1, Lilongwe, Malawi

**Keywords:** Utilization, Women of child-bearing age, Cervical cancer screening

## Abstract

**Background:**

Cervical cancer screening (CCS) uptake remains low in poor countries. Few studies have assessed individual need and health system factors which facilitate/impede use of healthcare services, including CCS utilization. Thus, we examined associations between these factors and CCS utilization among women of child-bearing age (WCBA) in Mangochi, Malawi.

**Methods:**

A cross-sectional study, sampling 482 women (18–49 years) using a multi-stage sampling method was conducted in five health facilities (HFs). Data were collected using a structured interview questionnaire from June-July, 2019. Chi-squared or Fisher’s exact tests were used to compare the distribution of CCS utilization according to different independent groups.

**Results:**

Our study found that 13.1% of the study participants had a history of CCS. The proportion of WCBA with a history of CCS was significantly higher among HIV + women than HIV- women and women with unknown HIV status, respectively [27.3% (33/121) vs. 8.5% (30/353) vs. 0% (0/8), χ2 = 29.18, df = 2, *p* < 0.001]. Significantly higher among those who had ever heard of cervical cancer (CC) than those who had not [23.0% (60/261) vs. 1.4% (3/221), χ2 = 49.28, df = 1, *p* < 0.001], among those who heard of CC from HFs than those who heard through radios, friends/family and other sources, respectively [31.2% (44/141) vs. 16.7% (7/42) vs. 9.3% (5/54) vs. 16.7% (4/24), χ2 = 12.62, df = 3, *p* = 0.006], among those with positive beliefs towards CCS than those with negative beliefs [19.2% (53/276) vs. 4.9% (10/206), χ2 = 21.37, df = 1 *p* < 0.001], among those recommended for CCS by health workers (HWs) than those not recommended [19.6% (53/270) vs. 4.7% (10/212), χ2 = 23.24, df = 1, *p* < 0.001], among those willing to be screened by male HWs than those unwilling [14.4% (60/418) vs. 4.7% (3/64), χ2 = 4.57, df = 1, *p* = 0.033]. Fisher’s exact test showed that CCS uptake among WCBA varied significantly by level of knowledge of CC signs/symptoms, with 66.7% (12/18) and 19.8% (48/243) among those with high-level and low-level knowledge screened, respectively (*p* < 0.001).

**Conclusions:**

HIV status, ever heard of CC, sources of information, knowledge of CC signs/symptoms, beliefs, recommendations by HWs for CCS, willingness to be screened by male HWs were associated with CCS utilization. Thus, sensitization campaigns for CCS should be conducted to increase uptake. Further, health facilities should intensify health education on CC, including signs and symptoms to increase knowledge. In addition, CC program implementers should be willing to train both males and females to offer CCS as the clients are open to be attended to by male providers as well.

## Background

Women from sub-Saharan Africa have low utilization of cervical cancer screening (CCS), estimated at 12.87% although evidence suggests that cervical cancer (CC) is a global public health concern [1]. The CCS programmes face a number of challenges in these poor resource settings. For example, a low CCS uptake was reported among rural Zimbabwean women where only 9% of the respondents in a study had ever had CCS [2]. Likewise, Malawi CCS coverage in 2020 was low at 25.5% even though the target was to screen 70% of eligible women [3]. In Northern Ethiopia, a study established that 19.8% of age eligible women had ever been screened for CC [4]. The lower CCS utilization rate in low and middle income countries has been attributed to various factors, including accessibility to testing facilities, lack of health education, low socioeconomic status, low perceived risk of disease, fear of CC diagnosis, fear of pain and embarrassment, lack of female health care providers, busy schedules, and beliefs that such tests are unnecessary [5].

Malawi started screening for CC in the early 1980s through a donor-funded programme which phased out due to sustainability problems [6]. Later in 1999 a re-launch of the CCS programme was introduced as a pilot program which was thereafter scaled up in 2002. Later on, the Ministry of Health (MoH) through the Reproductive Health Directorate (RHD) formulated the National Sexual and Reproductive Health and Rights (SRHR) policy to integrate CC as an SRHR priority area [7]. In 2013, the Cervical Cancer Control Program (CECAP) started conducting a pilot study on the Human Papilloma Virus (HPV) vaccine [8]. In 2016, the National Cervical Cancer Control Strategy (2016–2020) was developed to guide the implementation of CC control activities by CECAP and other stakeholders [8]. The Southern Africa Litigation Centre report also affirms that lack of awareness of the disease by the general public as well as health workers is a contributing factor towards the low uptake of services and hence the high prevalence and mortality in Southern Africa [9].

Malawi has the highest mortality related to CC, with 51.5 deaths per 100,000 women per year and prevalence estimated at 72.49 cases per 100,000 women per year [10]. Nevertheless, evidence has shown that there are several factors affecting CCS utilization other than lack of knowledge of the disease or any other parameter related to it. For example, there are health system enabling and individual need factors that play a key role in utilization of health services, including CCS uptake among WCBA. A Kenyan study found that out of 85.2% of women who were recommended by medical personnel to go for CCS, only 46.3% did undertake the screening test [11]. These findings are similar to a Malawian study conducted in Blantyre district where 72.4% of the participants had heard of CCS but only 13.2% had gone for CCS [12]. This result was similar to what was found in another Malawian study done in Mangochi district which reported 13.1% CCS uptake among WCBA [13]. Similar CC studies conducted in southern Malawi showed that age, multiple sex partners, lack of husband’s approval for screening, lack of knowledge of the disease and screening services and distance to a facility were statistically significantly associated with the utilization of CCS services. Some of these factors contributed to delays in accessing the screening services [6, 12, 14, 15]. Further, a systematic review on barriers affecting uptake of CCS in low and middle income countries found that unfriendly or male work staff barred women from undergoing CCS [5]. Mangochi district mirrors national challenges in terms of CCS uptake. For instance, the providers could see 2 or 3 women with VIA positive result each day at one CCS clinic in the district in 2015 [6]. This was a pointer to the magnitude of the problem of CC in the district. Despite this realization, CCS remains a challenge. By 2016, Malawi had 154 facilities offering CCS services [16]. Of which, 14 facilities offering CCS were from Mangochi district and they were government, Christian Health Association of Malawi (CHAM) and private-owned [16]. Limited studies have been conducted to examine associations between individual need and contextual enabling factors and CCS utilization among WCBA. We applied the sixth revised version of Andersen’s Behavioral Model of Health Services Use which indicates that health behaviours are influenced by both contextual and individual characteristics and within these characteristics are the three dynamics namely; predisposing, enabling and need factors [17]. The Model predicts one’s use of healthcare services (CCS) by focusing on contextual enabling (health system) factors that facilitate or hinder the utilization of healthcare services, and one’s (individual) perceived or influenced need for care [17]. Thus, we examined the utilization of cervical cancer screening and its associated factors, both individual need and health system factors, among WCBA in Mangochi district, Malawi. The identified factors will help health authorities and their partners to implement a CC control programme that will improve the utilization of the CCS services and ensure early diagnosis and treatment among WCBA in the district.

## Materials and methods

### Study design and setting

This was a facility-based cross-sectional study design and the study was done in Mangochi, Malawi [13].

### Study participants

The study participants were WCBA attending health facilities [13].

### Sample and procedures

482 WCBA were sampled by applying probability proportional to size procedures [13].

### Data collection

Data were collected through a survey method using a paper-based structured interview questionnaire. The data collector was a registered nurse who had a training on the questionnaire prior to its use. During data collection, skip patterns were observed. For instance, those respondents who answered “no” to the question “ever heard of cervical cancer” in the knowledge section, the whole section was skipped and questioning continued in the other section. For example, the respondents were in the “access to CCS” section asked if they have ever undergone CCS regardless of them having knowledge or not. This was still asked in order to establish if respondents were getting the CCS without being informed (to check if information is given prior to offering the screening).

### Questionnaire, independent and dependent variables

In this study, the dependent variable was utilization of CCS. Respondents were asked if they had ever been screened for CC and the response was binary (yes − 1 or no − 0). For independent variables, we assessed both categorical and continuous data. Variables with continuous data were categorized accordingly. The respondents were asked questions pertaining to the health system factors; willingness to be screened for CC by male health workers, distance travelled from their respective villages to the facilities offering CCS and recommendation given by health workers for CCS as well as individual need factors; HIV status, history of multiple sex partners, had participants ever heard of CC, source of CC information, knowledge of signs and symptoms of CC, knowledge of CC risk factors and beliefs towards CCS.

## Key variables and measurements

### I. Individual need factors

The following were the variables that were examined under individual need factors; HIV status, life time sex partners, participants ever heard of CC, source of CC information, level of knowledge of CC signs and symptoms, level of knowledge of risk factors, level of beliefs towards CCS.

#### HIV status

HIV status was categorized and coded as below; HIV negative [HIV-] (1) which refers to respondents who did not have HIV as confirmed by HIV testing. HIV positive [HIV+] (2) which refers to respondents who had HIV as confirmed by HIV testing and Unknown HIV status (3) which refers to respondents who were unaware of their HIV status.

#### Life time sex partners

Life time sex partners was categorized and coded as below; with 2 or more sex partners (1) and those without or with 1 life time sex partner (2).

#### Had participants ever heard of CC?

This was categorized and coded as below; yes (1) and no (2).

#### Source of CC information

Source of CC information was categorized and coded as below; health facility (1), radio (2), friends/family (3) and other [television, newspapers/magazines, school/learning institution, & mobile public address system](4).

#### Level of knowledge of CC signs and symptoms

Level of knowledge was categorized as low-level – 1 and high-level – 2. A scoring method was developed. Knowledge about signs and symptoms of CC was measured by using a question with five knowledge answers. A total of 5 points were given to respondents who upon probing had given all the correct answers. Respondents were asked to mention CC signs and symptoms. The correct answers were; post coital bleeding (1 point), foul vaginal discharge (1 point), painful sex (1 point), lower abdominal pain (1 point) and abdominal mass as a sign of CC (1 point). A mean score of **0.64** was calculated. Respondents with scores above the mean score were deemed as having high knowledge of CC signs and symptoms whereas those with scores below it were deemed as having low knowledge [18]. Thus, even respondents with one correct answer were deemed as having high-level knowledge of CC signs and symptoms. We, therefore, considered a score of mean value or above **(≥ 3)** to this question as a high-level of knowledge, otherwise lower scores were deemed as low-level of knowledge of CC signs and symptoms [19].

#### Level of beliefs towards CCS

Level of belief was categorized as positive belief – 1 and negative belief – 2. A scoring method was developed. A total of 4 points were given to respondents who had given all the correct answers. Four statements were read to the respondents to gauge their beliefs towards CCS, namely; I am at risk of getting cervical cancer hence I need to go for screening (respondents were awarded 1 point if they gave either of the two responses – strongly agree or agree and 0 point if they gave strongly disagree and disagree as responses). Cervical cancer screening is important (respondents were awarded 1 point if they gave either of the two responses – strongly agree or agree and 0 point if they gave strongly disagree and disagree as responses). Cervical cancer is curable if diagnosed early (respondents were awarded 1 point if they gave either of the two responses – strongly agree or agree and 0 point if they gave strongly disagree, disagree and “not sure” responses). And the last one was, I am afraid the screening procedure might be painful that is why I have not gone for screening (respondents were awarded 1 point if they gave either of the two responses – strongly disagree or disagree and 0 point if they gave strongly agree, agree and “not sure” as responses).

A mean score of **2.64** was calculated. Respondents with scores above the mean score were deemed as having positive beliefs towards CCS whereas those with scores below it were deemed as negative beliefs towards CCS [18].

#### Level of knowledge of risk factors

Level of knowledge was categorized as high-level – 1 and low-level – 0. A scoring method was developed. Knowledge about risk factors of CC was measured by using a question with seven knowledge answers. A list of CC risk factors was read out loud to the respondents and they were asked if they knew them. The respondents had to indicate yes, no or do not know. Answering yes was assigned 1 point. A total of 7 points were given to respondents who had given all the correct answers. The CC risk factors were; having multiple sexual partners (1 point), STIs history (1 point), being HIV+ (1 point), early onset of sexual activity (1 point), family history of CC (1 point), having uncircumcised male partner (1 point) and high parity (1 point). A mean score of **5.62** was calculated. Respondents with scores above the mean score were deemed as having high knowledge of risk factors of CC whereas those with scores below it were deemed as having low knowledge [18].

### II. Health system factors

Three variables were assessed under health factors, namely; recommendations for CCS given by health workers, willingness to be screened for CC by male health workers and distance travelled to the health facility.

#### Recommendations for CCS given by health workers

Recommendations for CCS given by health workers was categorized and coded as below; yes (1) and no (2).

#### Willingness to be screened for CC by male health workers

Willingness to be screened by male health workers was categorized and coded as below; yes (1) and no (2).

#### Distance to the health facility

Data in terms of distance travelled by respondents from their respective villages to health facilities were collected by asking them to name the village where they came from. The Research Assistant (data collector) had estimated distances for all villages to the health facilities where the study was conducted. The estimated distances from health facilities to all villages were collected from the Environmental Health Department of Mangochi District Hospital. Data on distance were collected as a continuous variable and were later categorized and coded into the following; ≤10 km meant distance travelled by equal to or less than 10 km (1), 11–20 km meant distance travelled ranging from 11 to 20 km (2) and ≥ 21 km meant distance travelled by a respondent equal to or beyond 21 km (3).

### Statistical analysis

Data were entered and analyzed using Statistical Package for Social Sciences (SPSS) Inc. PASW Statistics for Windows, Version 18.0. Chicago: SPSS Inc. Chi-squared or Fisher’s exact tests were used to compare the distribution of CCS uptake according to different independent groups, and statistical significance was considered at *p* < 0.05.

## Results

Table 1 shows individual need factors associated with CCS utilization among WCBA in Mangochi district. The proportion of WCBA with a history of CCS was significantly higher among HIV + women than HIV- women and women with unknown HIV status, respectively [27.3% (33/121) vs. 8.5% (30/353) vs. 0% (0/8), χ2 = 29.18, df = 2, *p* < 0.001]. Significantly higher among those who had ever heard of CC than those who had never heard [23.0% (60/261) vs. 1.4% (3/221), χ2 = 49.28, df = 1, *p* < 0.001], among those who had heard of CC from health facilities than those who had heard through radios, friends/family and other information sources, respectively [31.2% (44/141) vs. 16.7% (7/42) vs. 9.3% (5/54) vs. 16.7% (4/24), χ2 = 12.62, df = 3, *p* = 0.006], among those with positive beliefs towards CCS than those with negative beliefs [19.2% (53/276) vs. 4.9% (10/206), χ2 = 21.37, df = 1 *p* < 0.001]. Fisher’s exact test showed that CCS uptake among WCBA varied significantly by level of knowledge of CC signs/symptoms, with 66.7% (12/18) and 19.8% (48/243) among those with high-level and low-level knowledge screened, respectively (*p* < 0.001). We, however, found that there were statistically no significant differences in CCS uptake between respondents with one or without a life time sex partner and those with two or more life time sex partners [9.6% (19/197) vs. 15.4% (44/285), χ2 = 3.44, df = 1, *p* = 0.064] as well as those with high and low knowledge of CC risk factors [25.7% (49/191) vs. 15.7% (11/70), χ2 = 2.86, df = 1, *p* = 0.091], respectively.

**Table 1:** Individual need factors associated with cervical cancer screening utilization among women of child-bearing age in Mangochi district

Factors	Utilization of cervical cancer screening		Total N = 482	χ2	df	P-value
	**Yes** **N = 63**	**No** **N = 419**				
HIV status				29.18	2	**< 0.001**
HIV-	30 (8.5)	323 (91.5)	353			
HIV+	33 (27.3)	88 (72.7)	121			
Unknown	0 (0.0)	8 (100)	8			
Sex partners in life time				3.44	1	0.064
0 or 1	19 (9.6)	178 (90.4)	197			
≥ 2	44 (15.4)	241 (84.6)	285			
Ever heard of CC				49.28	1	**< 0.001**
Yes	60 (23.0)	201 (77.0)	261			
No	3 (1.4)	218 (98.6)	221			
Beliefs towards CCS				21.37	1	**< 0.001**
Positive	53 (19.2)	223 (80.8)	276			
Negative	10 (4.9)	196 (95.1)	206			
	** N = 60**	** N = 201**	** N = 261**			
Source of CC information*				12.62	3	**0.006**
Health facility	44 (31.2)	97 (68.8)	141			
Radio	7 (16.7)	35 (83.3)	42			
Friends/family	5 (9.3)	49 (90.7)	54			
Other	4 (16.7)	20 (83.3)	24			
Level of knowledge of CC signs and symptoms*						**< 0.001**†
High-level	12 (66.7)	6 (33.3)	18			
Low-level	48 (19.8)	195 (80.2)	243			
Level of knowledge of risk factors*				2.86	1	0.091
High-level	49 (25.7)	142 (74.3)	191			
Low-level	11 (15.7)	59 (84.3)	70			

†Fisher’s exact test was used for analysis as appropriate

Note: P-values marked in bold indicate factors that are statistically significant.

*these questions were asked only to those who have ever heard of cervical cancer.

Table 2 shows the health system factors associated with CCS utilization among WBCA in Mangochi district. The proportion of WCBA who had done CCS was significantly higher among those recommended for CCS by health workers than those who were not [19.6% (53/270) vs. 4.7% (10/212), χ2 = 23.24, df = 1, *p* < 0.001]. Significantly higher among those willing to be screened by male health workers than those not willing [14.4% (60/418) vs. 4.7% (3/64), χ2 = 4.57, df = 1, *p* = 0.033]. We, however, found that there were statistically no significant differences among distances travelled to health facilities and CCS utilization among respondents [14.6% (56/383) {≤10 km} vs. 9.5% (7/74) {11-20 km} vs. 0% (0/25) {21-35 km}, χ2 = 5.42, df = 2, *p* = 0.067].

**Table 2:** Health system factors associated with cervical cancer screening utilization among women of child-bearing age in Mangochi district

Factors	Utilization of cervical cancer screening		Total N = 482	χ2	df	P-value
	**Yes** **N = 63**	**No** **N = 419**				
Recommendations for CCS given by health workers				23.24	1	**< 0.001**
Yes	53 (19.6)	217 (80.4)	270			
No	10 (4.7)	202 (95.3)	212			
Willingness to be screened by male health workers				4.57	1	**0.033**
Yes	60 (14.4)	358 (85.6)	418			
No	3 (4.7)	61 (95.3)	64			
Distance to health facility				5.42	2	0.067
≤ 10 km	56 (14.6)	327 (85.4)	383			
11–20 km	7 (9.5)	67 (90.5)	74			
≥ 21 km	0 (0.0)	25 (100.0)	25			

Note: P-values marked in bold indicate factors that are statistically significant

## Discussion

This study was aimed at examining the associations between individual need factors as well as the health system factors and CCS utilization among WCBA. The individual need factors focuses on how people view their own general health as well as professional judgement and test results they get and how these lead to a decision to seek medical care or not whereas the health system factors have an impact on facilitating or impeding healthcare service use [17]. For instance, WCBA are either motivated or demotivated by these factors to undergo CCS.

### Individual need factors associated with CCS utilization

We found that a proportion of respondents who had done CCS was significantly higher among HIV positive women than HIV negative women and women with unknown HIV status. This was in agreement with an Ethiopian study that suggested that women who had tested HIV positive were 5.6 times more likely to screen for CC than those who had tested HIV negative [4]. Similar findings were shared in a study done by [20] which found that patients who were living with HIV were almost 2 times more likely to screen for CC than the other patients who were HIV negative. A lot of the respondents at Mangochi district hospital were exposed to information on the link between HIV and CC as health education and screening is offered on daily basis to all HIV positive clients accessing services at the ART clinic. The national CECAP strategy also recommends that 80% of women on ART should be screened for CC [16]. Our finding, therefore, is also good feedback to the integration of HIV and CC services program as this will potentially improve uptake of CCS if scaled up across the district. We also found that respondents who had undergone CCS were significantly higher among those with general knowledge on CC (who had heard of CC) than those who had never heard of CC. Several studies have found a significant association, that women with knowledge on CC were more likely to screen for CC than those without knowledge of the disease [21, 22]. However, we observed that 1.4% (3/221) of the respondents who had never heard of CC had undergone CCS. This finding was interesting because we never expected that the respondents who had never heard of CCS would have ever been screened. We speculate that other women might have just followed their colleagues recommended to undergo CCS and also underwent the screening without receiving health education or proper explanation. Whatever the case, we encourage health workers to provide comprehensive information to their clients before offering health services. Further, health workers should continue providing health education on CC to more women in the district to increase CCS utilization. This study found that the respondents who had been screened for CC were significantly higher among those whose source of information on CC was the health facility than those who whose source of CC information were radio, friends/family or other sources. Similar findings were shared in Namibian and Ethiopian studies where respondents who got information on CC from the health facilities were more likely to undergo CCS [23, 24]. Another Malawian study also found that women expressed that they had first heard the CC information from health workers (35%), relations or neighbors (34%) and from the radio (30%) [13]. This disagrees with a Ghanaian study which suggested that the media was crucial in influencing women to go for CCS and media types highlighted that motivated women to be screened for CC were radio and television [25]. Other studies have also highlighted the critical role that media plays in providing CCS information to the masses. For instance, a high screening prevalence has been reported in women who had media exposure in Kenya [26] and Nigeria [26]. Another Malawian study done in Phalombe district also indicated that radios were the main source of information followed by health workers among men [27]. This current study finding shows how important it is for the health workers to provide information on CC to women at every opportunity they present themselves to the health facility. Our study also found that proportion of respondents who had done CCS was significantly higher among those with high-level knowledge of CC signs and symptoms than those with low-level. Similar findings were reported by another Malawian study done by [12]. However, a Kenyan study by [20] found that women who did not know that bleeding after sex is a sign of CC had increased chances of accepting a CCS test than those who knew [20]. Our study had a lot of women with low level of knowledge about the signs and symptoms of CC. This entails that health education or information giving on CC should be detailed, covering all areas including signs and symptoms of CC as being more knowledgeable can result into increased uptake of CCS. Further, our study found that CCS was significantly higher among those with positive beliefs towards CCS than those with negative beliefs. These results are similar to results from Ethiopian and Malaysian studies [4, 18, 24, 28]. Equally, the behavioral model of health services use states that for one to undertake a personal health practice, the practice is influenced by a perceived need which is described as how people view their own general health and function status [17]. Thus, the perceived need is what influences the decision of whether or not one should seek medical care.

### Health system factors associated with CCS utilization

Our study suggested that a proportion of respondents who had done CCS was significantly higher among those who were recommended for CCS by health workers than those who were not. This is in agreement with findings from a Kenyan study that reported that health education or advice given by health workers was statistically significantly associated with uptake of CCS services [11]. Further, the same Kenyan study found that below half (46.3%) of the women had undergone CCS although over three-quarters of women (85.2%) were recommended for CCS [11]. These findings, notwithstanding, it is important, however, that health workers should intensify health education as well as giving proper advice in terms of CCS to women to improve CCS utilization in Mangochi district.

Our results established that a proportion of respondents who did CCS were significantly higher among those willing to be screened for CC by male health workers as compared to those who were not willing. This was a unique finding in a Muslim dominated setting where modesty is paramount. According to health workers in a Malawian study, the use of male service providers in CCS clinics was a barrier in provision of services in the country [6]. Further, the health workers had noted that the clients preferred older female service providers. Findings from another Malawian study done in Phalombe district with married men reported that most men (77%) had no problem with the gender of the health worker conducting the CCS [27]. Men are decision makers in Malawian culture as it is with most African cultures and their approval of both male and female service providers may ease the woman’s decision making to undergo CCS. This was different from studies done elsewhere in Africa where women expressed that age and gender of the service provider was a determining factor for them to go for the CCS or not [11]. Further, men indicated that they would not allow their women to go for CCS if the health provider is male stating that it was a taboo for another man to see their women’s private parts except during child birth. It is, therefore, very necessary to consider the cultural requirements in different communities and personal preferences of women when performing CCS. Our assumption in this study is that WCBA are familiar with male service providers when accessing other services aside from CCS hence this finding.

### Limitations of the study

Our study had its own limitations which included the following; firstly, data collectors read out the list of risk factors for CC and list of beliefs towards CCS which might have run the risk of women giving socially desirable responses [13]. Furthermore, this study was done in the health facilities as a result we might have missed views from women who were at home during the time we conducted data collection.

## Conclusions

Distance travelled to health facilities, number of sex partners in life time and knowledge of CC risk factors were not statistically significantly associated with CCS utilization among WCBA. Recommendations by HWs for CCS, willingness to be screened by male HWs, HIV status, beliefs, had participants ever heard of CC, knowledge of CC signs and symptoms, sources of information were statistically associated with CCS utilization. Thus, sensitization campaigns for CCS should be conducted to increase utilization. Further, health facilities should intensify health education on CC, including signs and symptoms to increase knowledge among WCBA and CC program implementers should disregard gender when training CCS providers as the clients are open to be attended to by male providers as well.

## Data Availability

The datasets generated during and/or analyzed during the current study are available from the corresponding author on a reasonable request.

## List of abbreviations

CC: Cervical Cancer
CCS: Cervical Cancer Screening
CECAP: Cervical Cancer Control Programme
CHAM: Christian Health Association of Malawi
DHO: District Health Office
HIV/AIDS: Human Immuno-deficiency Virus/Acquired Immuno-deficiency Syndrome
VIA: Visual Inspection using Acetate Acid
WBCA: Women of Child-Bearing Age
